# Hepatic Surgery Facilitated by a New Jet Dissector

**DOI:** 10.1155/1991/60679

**Published:** 1991

**Authors:** H. U. Baer, G. J. Maddern, L. H. Blumgart

**Affiliations:** Department of Visceral and Transplantation Surgery Inselspital University of Berne Berne Ch-3010 Switzerland

## Abstract

Increasing experience with major hepatic resections has stimulated the development of improved resectional techniques and tools. A new high velocity water jet dissector is reported which offers significant advances over previously developed ultrasonic and low pressure water jet machines. It has been successfully used in 8 major hepatic resections with minimal blood loss, excellent visibility and without complications. The dissector is also of value in the exposure of intrahepatic bile ducts for biliaryenteric anastomosis.


*HPB Surgery*, 1991, Vol. 4, pp. 137–146
Reprints available directly from the publisher
Photocopying permitted by license only
© 1991 Harwood Academic Publishers GmbH
Printed in the United Kingdom

### HEPATIC SURGERY FACILITATED BY A NEW JET DISSECTOR

H.U. BAER, G.J. MADDERN and L.H. BLUMGART
*Department of Visceral and Transplantation Surgery, Inselspital, University of Berne, Ch-3010 Berne, Switzerland*

*(Received 20 January 1991)*

Increasing experience with major hepatic resections has stimulated the development of improved resectional techniques and tools. A new high velocity water jet dissector is reported which offers significant advances over previously developed ultrasonic and low pressure water jet machines. It has been successfully used in 8 major hepatic resections with minimal blood loss, excellent visibility and without complications. The dissector is also of value in the exposure of intrahepatic bile ducts for biliary-enteric anastomosis.

KEY WORDS: Hepatic resection, water jet, bilioenteric anastomosis

### INTRODUCTION

Major hepatic resection for a variety of conditions, and in particular for the management of benign and malignant liver tumours, can now be carried out with an acceptable mortality and morbidity. Recent trends include the performance of more major resections, especially for large liver tumours, and the development of techniques for segmental resection and for resection in patients with cirrhotic livers.

The mortality amd morbidity of hepatic resection is largely due to haemorrhage, either during or after operation and the major intraoperative problem is control of haemorrhage. In particular while haemorrhage arising from the hepatic artery or portal vein is usually easily controlled, hepatic venous haemorrhage remains a threat, especially for large tumours encroaching on the vena cava or hepatic veins.

All methods of hepatic parenchymal transection are directed towards isolation and control of the vascular elements arising from the portal pedicles and of the major veins draining the liver into the vena cava. Methods of gaining such control include extrahepatic dissection of the elements of the portal pedicles, pedicular control, retrohepatic and suprahepatic control of the hepatic veins or intraparenchymal dissection. Vascular isolation of the liver is practiced by some. Many surgeons use a combination of these techniques[1,2].

The most commonly used method of dividing the parenchyma is by the finger fracture method, originally described by Lin[3] but adapted usually by the utilization of a clamp to crush the parenchyma leaving vessels and bile ducts intact and

Address correspondence to: Dr. H.U. Baer, Klinik Für Viszerale und Transplantationschirurgie, Inselspital, 3010 Berne, Switzerland exposed for ligation. There have been many attempts to improve on this simple technique, Almersjo[4] and others[5] introduced a suction knife and Hodgson[6] reported experience with an ultrasound dissector which disrupts liver parenchyma by application of ultrasonic waves and isolates the vessels and bile ducts[7]. The latter technique has found wide application, especially for segmental resections but there are few comparative studies as to its efficacy compared to the traditional finger fracture technique. Experienced surgeons, however, find the method slow and that the instrument does not readily cut tissue with any degree of density. One study shows that the only difference when comparing the ultrasound dissector with finger fracture was that operation time was significantly prolonged when the ultrasound dissector was used[8].

A completely new approach, utilizing a commercially available water jet to disrupt the liver parenchyma and expose the vessels was introduced by Papachristou[9]. This method was further developed and refined by Persson[10] using a specifically designed machine and Uchino[11] (Table 1). However, these initial attempts suffered from the defect of employing a low pressure system with consequent impaired efficiency.

We have developed a high pressure, high velocity water jet instrument (Table 1) which offers a range of advantages over earlier methods of hepatic parenchymal dissection (ME Medical Exports AG, Normannenstr. 8, Berne, Switzerland). The purpose of this report is to detail this machine and to briefly refer its initial clinical application not only in major hepatic resection but also in the intrahepatic exposure of bile ducts for biliary enteric anastomoses in clinically difficult situations in which the well described methods of intrahepatic ductal exposure are hazardous or ineffective.

### *Equipment and Technique*

The instrument consists of an hydrodynamic device for the elimination of parenchymal tissue so as to expose vascular and ductal structures. The system comprises a pressurized liquid source feeding via a flexible hose to a hand piece from which a fine nozzle extends (Figure 1). The nozzle has a diameter of either 20 or 70 u.

**Table 1** Comparison of different waterjet models.

| *Characteristics* | *LPS Persson* | *LPS Uchino* | *HPSS Baer* |
|---|---|---|---|
| 1 Diameter of nozzle | 0,08–0,12 mm | 0,10 – 0,22 mm | 0,25 – 0,06 mm |
| 2 Pressure range | 15 bars–50 bars | 15 bars–18 bars | 200 bars–1000 bars |
| 3 Flow rate ml/sec | 0,5–0,7 | 0,5–1,8 | 0,1–0,5 |
| 4 Kinetic energy at nozzle (W) | 1,4 | 1,2 | 22 |
| 5 Distance of coherent jet stream (mm) | >100 | >100 | 30 |
| 6 Option to alter jet form | no | no | yes |
| 7 Velocity of jet | 80 m/s | 47 m/s | >320 m/s |
| 8 Formation of blood bubbles | yes | yes | no (destroyed by the high speed micro droplets) |

LPS = Low Pressure System
HPSS = High Pressure System
W = Watt

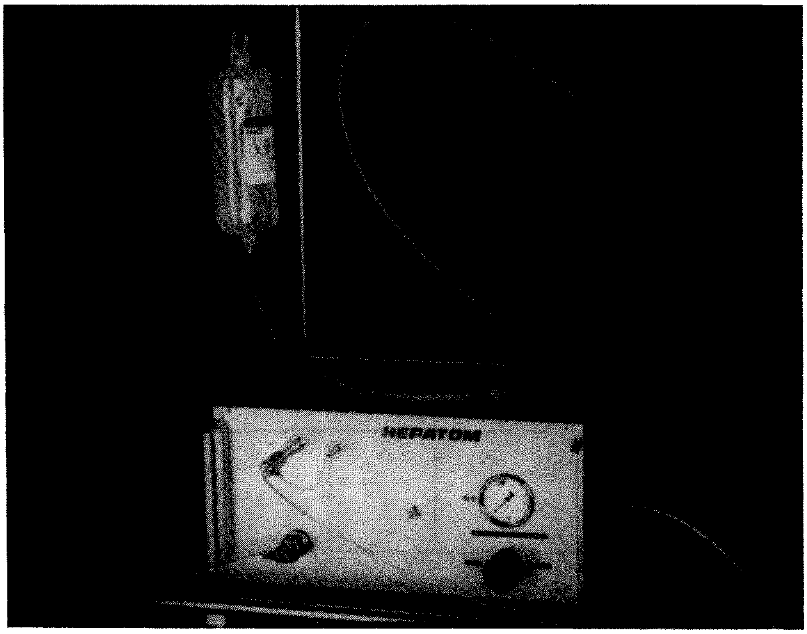

**Figure 1** Photograph of the assembled dissector showing sterile water bag, pump, connecting tubing and nozzle (reproduced with permission of the British J. of Surgery (in press)).

Water or saline is delivered at a pressure of 6 × 10,7 Pa allowing the stream flowing from the nozzle to have an initial velocity of at least 300 m/sec. (Figure 2). This high velocity jet increases in diameter as the distance from the tube increases. The jet stream is coherent for 30 mm from the tube tip and then radiates forming microdroplets and finally a spray of water only at a distance of 40–80 cm from the tip of the tube (Figure 3a). During operation the tip of the tube is advanced from a distance of about 40 cm towards the surface to be cut so that as the beam approaches increasing pressure is applied with disruption as the force of the jet becomes greater. During dissection of the parenchyma, as vascular structures become evident, the instrument is withdrawn. To and fro movements of the hand, thus varying the intensity of the jet, allow cleaning of vascular and ductal structures. Little experience is needed to allow effective use of the instrument. Once vessels are exposed they are either electrocoagulated, clipped or divided and ligated. Visibility in the operating field is excellent since the water jet washes away blood and parenchymal debris and the microdroplets remove air bubbles effectively.

The sterility of the system was assessed by contaminating all lines with a Proteus bacteria overnight. On the next day the lines were emptied and sterilized with Gigasept 10% (Butan 1–4–Dial/2,5, Dimethoxytetra-Hydrofuran, Winthrop, 8025 Zürich, Switzerland) for 10 minutes and the surface of the tubes sprayed with industrial alcohol spray 70%. The operating nozzle was gas sterilized. The entire system was flushed with sterile solution for 5 minutes prior to use. Samples collected at completion of this procedure showed no evidence of the Proteus bacteria on subsequent culture.

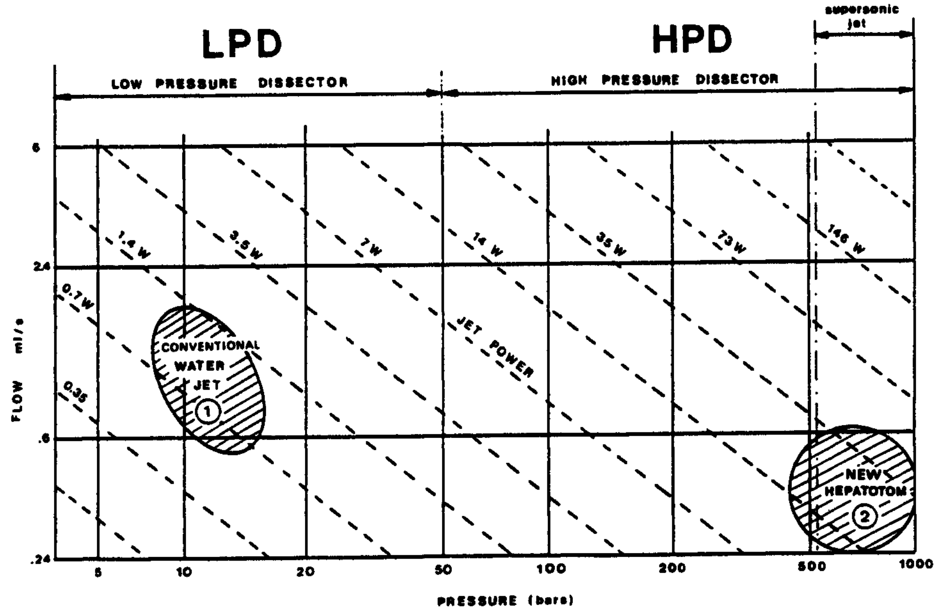

**Figure 2** The operating flow and pressure characteristics in which the conventional 1 and the new water-jet 2 (Hepatotom) operate (reproduced with permission of the British J. of Surgery (in press)).

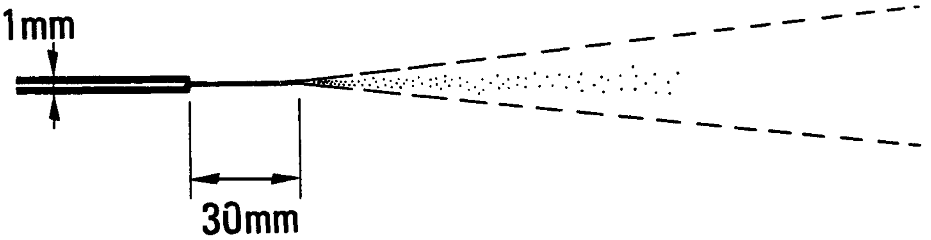

**Figure 3a** Diagramatic representation of the new jet dissector stream characteristics, showing a divergent stream after 30 mm with microdroplet formation.

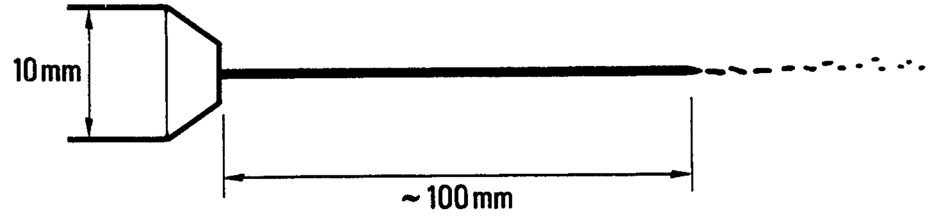

**Figure 3b** Diagramatic representation of conventional jet stream characteristics, showing a coherent stream for 100 mm and subsequent droplet formation (reproduced with permission of the British J. of Surgery (in press)).

### CLINICAL APPLICATION

There are a variety of clinical settings in which this machine might prove useful but we have confined ourselves in our initial experience to hepatobiliary surgery and in particular an application of the machine during liver resection and for the exposure of intrahepatic bile ducts for biliary-enteric anastomosis.

### *Liver Resection*

The system has been employed for 8 major liver resections (2 extended right hepatectomies, 1 right hepatectomy and 2 extended left hepatectomies, 1 left hepatectomy) and for 2 segmental resections including one patient requiring reoperation.

The method of hepatectomy employed has been previously described in detail[2]. For major resections initial control of the vessels supplying the area to be removed has been obtained at the hilus or within the umbilical fissure and control of the hepatic veins when possible has been obtained by retrohepatic dissection of the inferior vena cava. In three of these patients however, the extrahepatic veins could not be confidently displayed because of tumour proximity and in both of these instances the major hepatic veins were defined within the liver parenchyma. For extended left hepatectomy initial control of the hilar structures was followed by a parenchymal dissection commencing in the plane between segment V and VI at the inferior margin of the liver and extending upwards to display the structures of the right portal pedicle and in particular to define, close to the hilus, the anterior and posterior sectoral pedicles. The dissection was then continued upwards anterior to the vena cava so as to define the middle and left hepatic vein.

The parenchymal transection followed initial incision of Glisson's capsule and the new machine was used either on its own for parenchymal dissection or in combination with a finger fracture technique.

It was found that the new machine allowed rapid transection of the parenchyma with accurate display of intraparenchymal vascular and ductal structures (Figure 4) and that the machine was particularly helpful in defining the major veins draining the liver and especially in the definition of the right portal pedicle during extended left hepatectomy. The machine allows dissection of even quite firm and fibrous tissue when held close to the parenchyma.

In particular the machine was extremely useful where tumour came to lie close to major veins and this advantage was particularly evident in extended left hepatectomy and also in one of the segmental resections in which a recurrent tumour (following previous extended left hepatectomy) was found to lie in very close proximity to the remaining right posterior sectoral pedicle. Indeed in this case it was even possible to individually identify the vessels feeding the tumour and to ligate these separately.

The mean time for routine right hepatic resection or left hepatic resection in our department is approximately four hours and for extended left hepatectomy five hours. The number of cases in which we have tried using the new technique is not sufficient for full assessment but certainly there was not any prolongation of operating time. Blood loss for right or left hepatectomy or extended right hepatectomy averages some 500 ml for operations in which the tumour does not encroach on major vessels of the hilus or in the region of the vena cava but is somewhat higher for larger tumours in close proximity to these structures. The blood loss

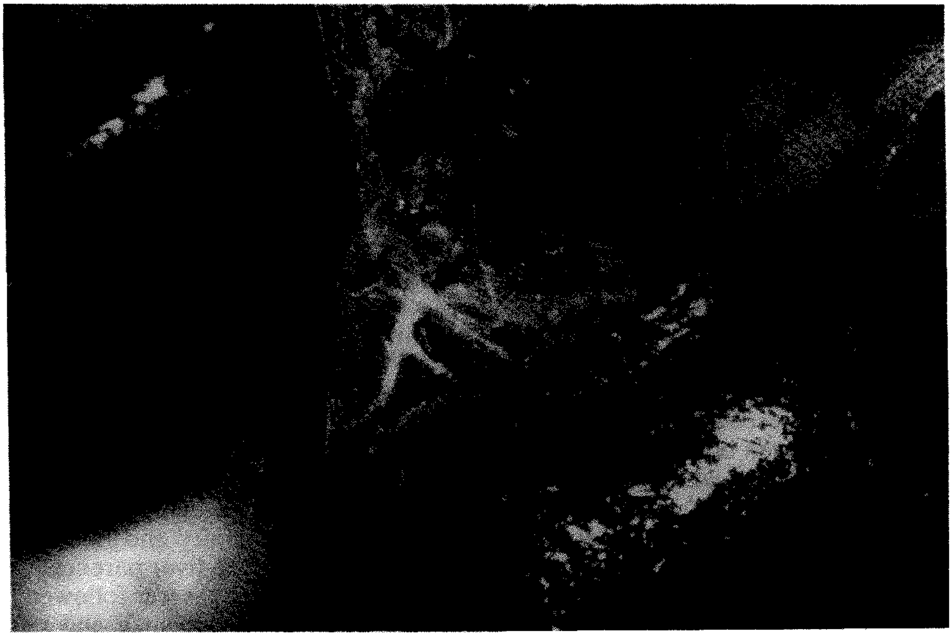

**Figure 4** Operative photograph showing jet of water displaying intraparenchymal structures which can then be safely clipped and ligated.

encountered during trial of the machine was within these limits except for extended left hepatectomy in which it is our initial impression that it was reduced and that operation was facilitated.

*Biliary-enteric Anastomosis*

Biliary-enteric bypass for malignant obstruction at the hilus of the liver can be effected by well established techniques using the ligamentum teres approach[12,13,14]. Other approaches include the Longmire-Sandford operation[2,15] which necessitates a removal of the peripheral part of segment II and III of the liver in order to gain access to the intrahepatic ducts. The former operation may not be possible where tumour is found to infiltrate to the base of the ligamentum teres or where an anatomical distortion caused by hypertrophy of the left liver in the presence of right liver atrophy makes access to the umbilical fissure difficult. The Longmire operation is not easily performed and is a relatively major procedure.

In such patients an alternative approach is to carry out a more limited removal of parenchyma either on the left or the right liver so as to expose an intrahepatic duct for anastomosis.

In four patients we have used the jet-dissector to create access for biliary-enteric anastomosis to intrahepatic bile ducts. In three of these patients hilar cholangiocarcinoma was extending to the left and required a segment III approach and in the fourth patient a cholangiolar hepatocellular carcinoma was causing high biliary obstruction, rendering convential approaches impossible. Percutaneous transhepatic intubation is used regularly in our department but was considered unsuitable in these patients.

The jet-dissector was used to expose an area of some 3 $cm^2$ on the lower surface of the left liver within segment III. It was found that the liver parenchyma could be gently washed away exposing a tree of peripheral branches of the segment III bile duct. It was possible to create a sufficiently wide anastomosis (2 cm length) to a peripheral duct. In one of these patients the approach was just to the left of the umbilical fissure in order to expose the main segment III duct as described by Soupault[12].

The procedure was accomplished with minimal blood loss and enabled an effective anastomosis to be performed.

### DISCUSSION

The new instrument has proved, during our initial experience, to be helpful and allow accurate exposure of major blood vessels so that they can be easily controlled. The instrument proved a valuable adjunct for the experienced surgeon in routine cases and especially useful for major hepatic resection where only minimal clearance from major vessels could be made in the resection of large tumours. The machine is rapid to use and the technique is easily learned. We have been using the ultrasonic dissector for some time and find the instrument useful but somewhat slow and it is not effective with more fibrotic tissue. Furthermore the ultrasonic vibrator hand piece is somewhat large and much more difficult to manipulate within a small space than is the water jet dissector. A comparison of the ultrasonic vibrator with the conventional finger fracture technique was published by Rau *et al.*, 1990[8] and the only difference found in the study related to the increased time taken to perform hepatic resection using the ultrasound dissector.

Our experience confirms the initial report of Papachristou[9] who employed the water jet machine and reported its use in four minor hepatic interventions. Persson[10] introduced a low pressure water jet system that produced a water jet with a coherent beam of about 100 mm (Figure 3b) and suggested the addition of vascoactive drugs in the perfusate to further increase its hemostatic properties. However the pressure applied could only be manipulated by altering the pressure in the pumping system. In comparison to these experiences the water jet dissector here reported offers a range of advantages. The small outlet of only 0.02 u and a specially designed nozzle produces a water jet which alters its pressure in accordance to the distance from the tip of the nozzle. The required pressure can therefore be easily adjusted by moving the hand to and fro. The flow of water is small, so that even in major extended hepatic resection the volume of fluid used is only 200–300 ml. The danger of absorption of fluid into the circulation is obviated. The production of microdroplets provides excellent visibility in the operating field. The speed of resection is almost comparable to that obtainable with the finger fracture technique and firm or even fibrous tissue can be dissected.

The jet indeed can be so adjusted, that liver parenchyma can be cleared to allow access to bile ducts for difficult intrahepatic biliary-enteric anastomosis.

Our initial experience with the new machine is encouraging and warrants further trial.

1. Bismuth, H. (1982) Surgical anatomy and anatomical surgery of the liver. *World J. Surg.*, **6**, 3–9
2. Nlumgart, L.H. (1988) Liver resection—liver and biliary tumours. In *Surgery of the liver and biliary tract*, edited by L.H. Blumgart, vol II, pp. 1251–1280. Edinburgh, London, Melbourne, New York: Churchill Livingstone
3. Lin, T.Y. (1973) Results in 107 hepatic lobectomies with a preliminary report on the use of a clamp to reduce blood loss. *Ann. Surg.*, **177**, 413–421
4. Almersjö, O. and Hafström, L. (1974) The "suction knife". A new device for dividing liver parenchyma. *Acta Chir. Scand.*, **140**, 581–583
5. Haapiainen, R. and Schröder, T. (1989) The suction knife in liver surgery. *Amer. J. Surg.*, **157**, 340–342
6. Hodgson, W.J.B. and DelGuercio, L.R.M. (1984) Preliminary experience in liver surgery using the ultrasonic scalpel. *Surgery*, **95**, 230–234
7. Putnam, CH.W. (1983) Techniques of ultrasonic dissection in resection of the liver. *Surg. Gynecol. Obstet.*, **157**, 475–478
8. Rau, H.G., Thomas, S., Arnold, H. and Schildberg, F.W. (1990) Schneiden mit dem Wasser-Strahl (Jet-Cutting an der Leberchirurgie—Jet-Cutting versus Ultraschall-Aspirator) In *Chirurgisches Forum 1990 f. experim. u. klinische Forschung*, edited by R. Häring *et al.*, pp. 419–426. Berlin, Heidelberg: Springer-Verlag
9. Papachristou, D.N. and Barters, R. (1982) Resection of the liver with a water jet. *Br. J. Surg.*, **69**, 93–94
10. Persson, B.G., Jeppsson, B., Tranberg, K.G., Roslund, K. and Bengmark, S. (1989) Transection of the liver with a water jet. *Surg. Gynecol. Obstet.*, **168**, 267–268
11. Uchino, J., Une, Y., Horie, T., Yonekawa, M., Kakita, A. and Sano, F. (1988) Surgical cutting of the liver by water jet. Sendai, 9th International Symposium on Jet Cutting Technology. BHRA, The Fluid Engineering Centre, Cranfield, Bedford U.K. pp. 629–639
12. Soupault, R. and Couinaud, C. (1957) Sur un procédé nouveau de derivation biliaire intrahépatique: la cholangio-jejunostomie gauche sans sacrifice hépatique. *Presse Med.*, **65**, 1157
13. Hepp, J. and Couinaud, C. (1956) L'abord et l'utilisation du canal hépatique gauche dans les réparations de la voie biliaire principale. *Presse Med.*, **41**, 947–949
14. Blumgart, L.H. and Kelley, C.J. (1984) Hepaticojejunostomy in benign and malignant high bile duct stricture: approaches to the left hepatic ducts. *Br. J. Surg.*, **71**, 257–261
15. Longmire, W.P. and Sandford, M.C. (1948) Intrahepatic cholangiojejunostomy with partial hepatectomy for biliary obstruction. *Surgery*, **128**, 330–347

*(Accepted by S. Bengmark on 20 January 1991)*

### INVITED COMMENTARY

It is always a pleasure to see that new technology continues to be applied to hepatic surgery. This is a tacit acceptance that in many hands finger fracture is bloody, uncontrolled and can be disastrous if a major vessel is torn. The ultrasonic dissector was the first technique which allowed the surgeon to routinely define hepatic anatomy in a controlled manner. It has been accepted widely and has been challenged consistently, but in many surgeon's hands, after a proper teaching program, it has been helpful. The application of liver clamps to control bleeding, most of which are variations of that described by Lin, do not seem to have found favor in the West. The Yag Laser was no more effective than electrocautery. The microwave approach was interesting but again no help in defining of anatomy. Different suction knives have been introduced from time to time but these variations on simple suckers have not led to controlled divisions of the liver. Previous versions of the water jet produced large volumes of water, some of which was sometimes injected into the hepatic vessels, have not found favor.

Baer and his colleagues made a common mistake in that it is incorrect to say that the ultrasonic dissector disrupts liver parenchyma by ultrasonic waves. In fact, it requires direct contact with parenchymal cells in order for its cavitating effect to occur. This gives tactile feedback to the surgeon who can spare the vascular and biliary tree. In our hands the ultrasonic dissector is not slow and I think it is faily common now for major hepatic lobectomies to be done in three to four hours with about 500 cc of blood loss using this technique.

There can be no tactile feedback with a water jet. This can lead to accidents such as perforation of the viscus if not properly aimed. Starting the procedure by holding the tip about two feet away from the liver, I would have thought could make aiming extremely difficult. From that distance it may not even be possible to hit the wound. I am also concerned that thin walled hepatic veins may easily be disrupted, particularly, if, as is claimed, the machine dissected fibrous tissue. On the other hand, I am pleased to note that the volume of water used by the new high velocity water jet does appear to have been reduced compared to the huge amounts previously required with this technique.

The ideal technique for hepatic parenchymal dissection would allow the surgeon to rapidly locate and ligate all large vessels; it would leave the divided surface of the liver sealed to prevent late bile or blood seepage; it would be repeatable so that the technique could be taught to surgical residents; it would allow the surgeon to accurately demonstrate hepatic anatomy intraoperatively. The finger fracture technique does not achieve these objectives. The ultrasonic dissector is close to achieving these objectives. It remains to be seen if the new water jet can achieve these objectives.

W. John B. Hodgson
New York Medical College
New York, USA

### INVITED COMMENTARY

Lobar liver resections as well as segmental and subsegmental liver resections are now performed with acceptably low mortality and morbidity. Most "one institution series" reports 5% mortality. The mortality is to a great extent related to per- or postoperative haemorrhage. In that respect, peroperative bleeding from major liver outflow veins in particular is the critical issue. The arteries and the branches from the portal vein are usually easy to dissect and control in the hepatic duodenal ligament or during intraparenchymal transection. When the tumours are close to or engulfing the hepatic veins or the retrohepatic cava a particularly complicated and risky procedure is at hand. The finger-fracture technique — digitoclasia, which was introduced in 1958 by Lin has since then been the standard technique for dividing the parenchyma. The method is usually rapid, but can be risky — tearing the intermediate sized vessels, particularly the veins.

A safer and more reliable method based on modern knowledge of liver anatomy is needed now that liver resection for cancer is becoming increasingly frequent and being performed by more surgeons.

During recent years there has been an overflow onto the market of new devices with the goal of providing controlled intraparenchymal liver dissection, good haemostasis and a clean operative field. These devices have different advantages. The ultrasound dissector, which supposedly is the post commonly used device, is considered by most liver surgeons to prolong surgery, but contributes increased safety. Whether the blood loss is decreased has not been properly investigated in controlled series. It has also been claimed that ultrasound dissection causes less tissue damage and thereby reduces the incidence of infections.

A recently introduced device using a water jet dissection to disrupt the liver parenchyma has now been improved by Blumgart and coworkers. They have changed the system to provide higher pressure and a higher velocity water jet, which evidently offers a range of advantages over the previous methods.

The new technique should be properly investigated by several liver surgeons. The technique was reported to be especially useful when the tumour was close to major hepatic veins. In this respect as well as of course in all other kinds of surgery the value of equipment employed is related to the experience of the surgeons involved. Nevertheless, all new devides to make liver surgery more safe must be most welcomed.

In conclusion it has to be stated that a mandatory demand for all new equipment is that its value is scientifically assessed with respect to improvement of results or increased safety.

L.O. Hafström
University of Gothenburg
Gothenburg
Sweden

